# Colonic actinomycosis presenting as a palpable colonic mass with normal colonic mucosa

**DOI:** 10.1093/jscr/rjab381

**Published:** 2021-09-08

**Authors:** Mai Charernsuk, Suppadech Tunruttanakul, Ratchanee Tunruttanakul, Borirak Chareonsil

**Affiliations:** Department of Surgery, Sawanpracharak Hospital, Nakhon Sawan, Thailand; Department of Surgery, Sawanpracharak Hospital, Nakhon Sawan, Thailand; Department of Surgery, Sawanpracharak Hospital, Nakhon Sawan, Thailand; Department of Surgery, Sawanpracharak Hospital, Nakhon Sawan, Thailand

## Abstract

Colonic actinomycosis is rare and can present as an ill-defined intra-abdominal mass that can be difficult to differentiate from colon cancer. This case report aims to share the details of this case and provide diagnostic clues. A 63-year-old female presented with a palpable right-sided abdominal mass. Computed tomography (CT) revealed irregular thickening of the colonic hepatic flexure, and colonoscopy detected no abnormalities. Five months later, the patient returned with an increase in the mass size. Repeat CT revealed lesion expansion, with suspected abdominal wall invasion. Extended right-hemicolectomy with abdominal wall wedge resection was performed, and the histological results were compatible with actinomycosis infection. Colonic actinomycosis is a rare chronic inflammatory disease. Normal colonic mucosa during colonoscopy, with clinical and imaging findings, may help physicians diagnose the condition preoperatively.

## INTRODUCTION

A palpable colonic mass is a worrisome clinical finding, and one of the differential diagnoses is invasive carcinoma. We report a case of cecal actinomycosis, which is a rare cause of a palpable colonic mass. Even with imaging and physical examination findings, it is challenging to differentiate this condition from cancer [1]. However, some clues may help clinicians in this ambiguous situation.

## CASE REPORT

**Figure 1:**
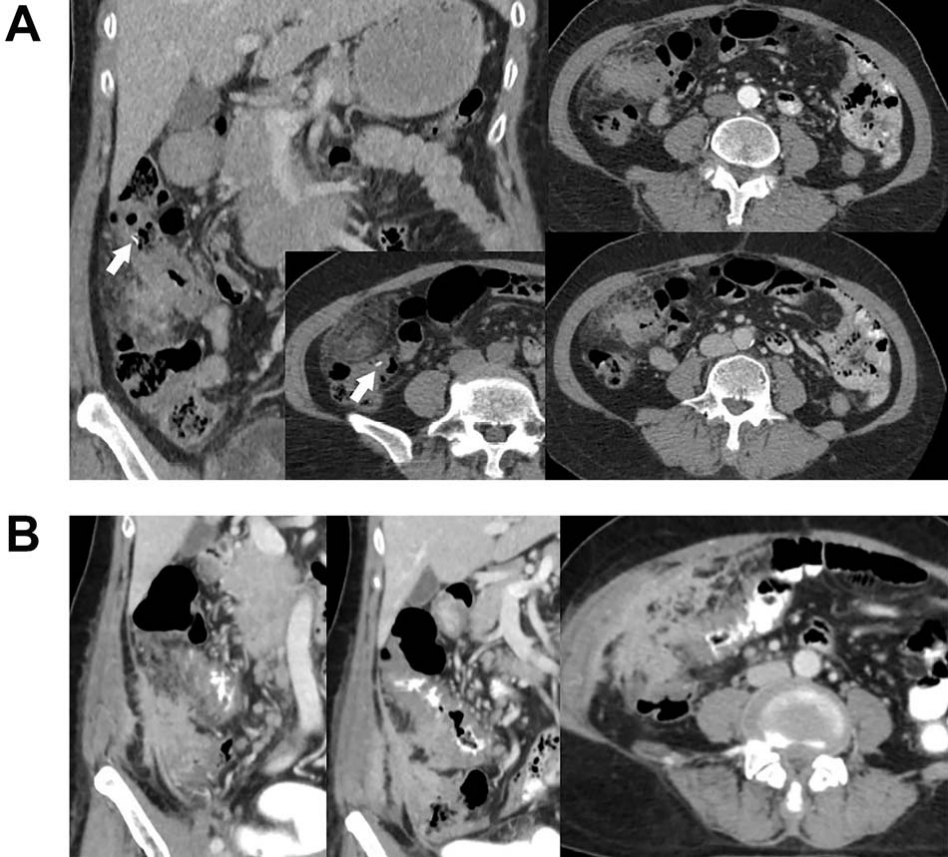
Contrast-enhanced abdominal CT. Images (**A**) and (**B**) were obtained 5 months apart. Image A shows irregular circumferential thickening of the colonic hepatic flexure. An elongated foreign body is also visible in the area (arrow). Image B shows an increase in the size and extension of the thickening. There is also a newly-detected heterogenous enhancing mass-like lesion involving the lateral wall of the cecum and proximal ascending colon, with enhancement indicating irregular thickening of the adjacent peritoneum.

A 63-year-old female with no underlying disease presented with a palpable mass on the right side of her abdomen. Abdominal computed tomography (CT) was performed at the primary hospital and revealed colonic hepatic flexure thickening (Fig. 1A). Interestingly, imaging also revealed a foreign body-like object near the affected area. The clinical significance of the particle was unknown. Colonoscopy was then performed to the terminal ileum, and no abnormalities were identified. A 1-month repeat CT scan was planned, but the patient refused and was lost to follow-up. The patient returned 5 months later with an increase in the size of the palpable mass, anorexia, weight loss and chronic intermittent low-grade fever. There were no bowel habit changes. On physical examination, her temperature was ~38.1°C, and slightly pale conjunctivae were noted; lymphadenopathy was not present. An ill-defined, at least 10-cm diameter, fixed, intra-abdominal mass was palpated in the patient's right lower abdominal quadrant. Blood test results were essentially normal except for mild anemia.

Repeat abdominal CT (Fig. 1B) revealed an increase in the size and extension of irregular circumferential thickening of the proximal transverse colon with a newly-detected heterogenous enhancing mass-like lesion involving the lateral wall of the cecum and proximal ascending colon. There was also irregular thickening enhancement of the adjacent peritoneum and right lower abdominal wall without other important organ invasion. The foreign body particles had disappeared. Cancer of the cecum was one of the differential diagnoses.

Although we believed that the problem was not cancer according to the colonoscopic findings, radical surgery was chosen to relieve the patient's symptoms, with acceptable risk owing to the lack of important organ invasion. After discussing surgery with the patient and her family, extended right hemicolectomy with wedge resection of the adjacent abdominal wall was performed as a cancer operation. There were no post-operative complications, and the patient was discharged on day five.

**Figure 2:**
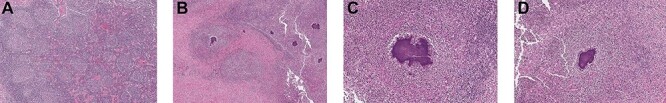
Histological images of the resected mass demonstrating multiple abscesses, necrosis and typical ‘sulfur granules’ in actinomycosis bacterial colonies.

The histological results indicated multiple abscesses with actinomycosis granulomas involving the pericolic fat, ascending colon and the resected abdominal wall. Figure 2 illustrates a typical actinomycosis colony with sulfur granules.

## DISCUSSION

Actinomycosis is a rare chronic inflammatory disease caused by the bacterial genus *Actinomyces*. Six of 14 species generate illness in humans, and the most common is *Actinomyces Israelii* [2]. The organism can generally inhabit the human gastrointestinal and female genital tract without inflicting disease (normal flora; [2]). Pathology hypothetically occurs when there is a mucosal membrane disruption from trauma or disease events leading to micro-organism invasion, even in immunocompetent individuals [3–5]. However, the actual mechanism changing the normal floral into pathogens is unknown [4]. Our patient had foreign body-like material within the disease area. This material, although inconclusive, might have caused a mucosal break, allowing entry for the organism. There is no person-to-person transmission and no external reservoir for these organisms [6].

The four clinical forms of actinomycosis are cervicofacial, thoracic, abdominopelvic and cerebral; the cervicofacial form is the most common [2]. Abdominopelvic actinomycosis is relatively rare and more commonly affects the ileocecal area [7]. However, the disease can develop in any location [8].

An intra-abdominal mass usually raises concerns for patients and physicians; mainly when the mass originates from the colon, as is often the case with cancer [9]. In our case, the differential diagnoses comprised tumors and chronic inflammatory diseases, and the normal colonic mucosa was a helpful clue to assist the diagnosis. Regarding tumors, colonic cancer with normal mucosa is very infrequent. Possible cancerous causes are tumors that do not originate from the mucosa, namely neuroendocrine tumors (NETs), lymphoma, gastrointestinal stromal tumor (GIST)/sarcoma and melanoma. Among these tumors, the most likely candidates are NETs and GIST/sarcoma, as both can have trivial endoscopic findings [10, 11]. The common CT findings for NETs are a homogeneously dense mass with homogenous intravenous contrast enhancement [12]. The CT findings for GIST/sarcoma range from colonic thickening to a noticeable colonic mass [11]. The potential colonic inflammatory diseases comprise inflammatory bowel diseases (IBD), diverticulitis, appendicitis and tuberculosis [2]. Again, normal colonoscopy findings provide a diagnostic clue to rule out IBD [13].

The confirmatory diagnosis of actinomycosis infection comprises demonstrating micro-organisms from pus or tissue biopsy; however these organisms are difficult to find [8]. CT-guided aspiration with or without a core-needle biopsy of the suspicious lesion has been reported for intra-abdominal pathologies [14] but can be performed only if cancer has been ruled out [15].

For complicated actinomycosis, a long course of 6–12 months of antibiotics is the treatment, and penicillin G is the drug of choice [2]. Interventions or even surgery may be required with abscesses, sinuses, the presence of necrotic tissue or masses with intestinal obstruction [4, 5]. To our knowledge, no previous reports involved only medical therapy to treat large mass-forming lesions [1, 5, 7]. Therefore, in our case, although the diagnosis was made preoperatively, surgery may have been inevitable. However, with a preoperative diagnosis, its radicality could have been decreased.

In conclusion, colonic actinomycosis is rare and difficult to diagnose. With clinical, imaging and colonoscopy findings, the differential diagnoses can be narrowed. Actinomycosis should be suspected in chronic inflammatory mass-forming lesions with normal colonoscopy findings.
